# Phage Resistance Reduced the Pathogenicity of *Xanthomonas oryzae* pv. *oryzae* on Rice

**DOI:** 10.3390/v14081770

**Published:** 2022-08-13

**Authors:** Mengju Liu, Ye Tian, Haitham E. M. Zaki, Temoor Ahmed, Rong Yao, Chengqi Yan, Sebastian Leptihn, Belinda Loh, Muhammad Shafiq Shahid, Fang Wang, Jianping Chen, Bin Li

**Affiliations:** 1 State Key Laboratory of Rice Biology, and Ministry of Agriculture Key Laboratory of Molecular Biology of Crop Pathogens and Insects, and Key Laboratory of Biology of Crop Pathogens and Insects of Zhejiang Province, Institute of Biotechnology, Zhejiang University, Hangzhou 310058, China; 2 Horticulture Department, Faculty of Agriculture, Minia University, El-Minia 61517, Egypt; 3 Applied Biotechnology Department, University of Technology and Applied Sciences-Sur, Sur 411, Oman; 4 Institute of Biotechnology, Ningbo Academy of Agricultural Sciences, Ningbo 315040, China; 5 University of Edinburgh Institute, Zhejiang University, Hangzhou 314400, China; 6 Fraunhofer Institute for Cell Therapy & Immunology (IZI), Department of Vaccines and Infection Models, Perlickstr. 1, 04103 Leipzig, Germany; 7 Department of Plant Sciences, College of Agricultural and Marine Sciences, Sultan Qaboos University, Al-khod 123, Oman; 8 State Key Laboratory for Managing Biotic and Chemical Threats to the Quality and Safety of Agro-Products, Key Laboratory of Biotechnology in Plant Protection of Ministry of Agriculture and Zhejiang Province, Institute of Plant Virology, Ningbo University, Ningbo 315211, China

**Keywords:** *Xanthomonas oryzae* pv. *oryzae*, gene, phage, pathogenicity, lipopolysaccharide

## Abstract

Plants grow together with microbes that have both negative and positive impacts on the host, while prokaryotes are in turn also hosts for viruses, co-evolving together in a complex interrelationship. Most research focuses on the interaction of either bacterial pathogens interacting with the plant host, or the impact on viruses on their pathogenic bacterial hosts. Few studies have investigated the co-evolution of bacterial pathogens with their host plants as well as with their bacterial viruses. In this work, we aimed to identify the genes that were associated with both phage sensitivity and host pathogenicity of the bacterium *Xanthomonas oryzae* pv. *oryzae* (Xoo), which is the most important bacterial rice pathogen. Using the Tn5 transposon mutation technology, we created a library of Xoo strain C2 comprising 4524 mutants, which were subsequently tested for phage infectability. The phage infection tests showed that less than 1% of the mutants (*n* = 36) were resistant to phage infection, which was attributed to the Tn5 insertion in 19 genes. Interestingly, three out of 19 genes that conveyed resistance to the phage resulted in reduced pathogenicity to rice seedlings compared to the wild type. We identified three genes involved in both phage infection and bacterial virulence, which were studied by knockout mutants and complementation experiments. All of the three knockout mutants were resistant to infection by phage X2, while the complemented strains restored the susceptibility to the bacterial virus. Surprisingly, the genes are also essential for pathogenicity, which we confirmed by single knockout mutants corresponding to the Tn5 mutants. All three genes are involved in lipopolysaccharide synthesis, thus changing the cell envelope surface molecule composition. Our work shows a possible balance in terms of the connection between bacterial virulence and phage resistance, supporting the deployment of phages for the biocontrol of plant pathogens.

## 1. Introduction

Bacterial blight caused by *Xanthomonas oryzae* pv. *oryzae* (Xoo) is the most important bacterial disease in rice, which can cause devastating yield loss and poor quality grain in rice-growing regions throughout the world, particularly in China [1,2]. Previous studies have found that the pathogenic mechanism of Xoo in host rice tissue depends on a variety of virulence factors, including extracellular enzymes, toxins, type III secretion system and its effectors, adhesion, lipopolysaccharide (LPS), extracellular polysaccharides, biofilm and cell movement, and others [3,4]. Rice pathogenic bacteria are also hosts for bacteriophages (phages). Bacteriophage therapy is considered as a novel strategy to control plant-based bacterial pathogens [5]. As a biological control agent, phages have many advantages due to self-replication, nontoxicity to eukaryotic cells, host specificity, ability to overcome resistance, and ease of production [6,7]. First, using phage instead of pesticides would reduce or even completely avoid environmental pollution by antimicrobial compounds and the pathogen resistance caused by chemical bactericides [8,9]. Second, phages are the most abundant organisms in nature, with an estimated number of 10^31^, which is about ten times the total number of prokaryotes [10].

Third, phages are highly specific and infect only suitable host bacteria, therefore they do not have a negative impact on other beneficial microorganisms, in contrast to chemical compounds used to control bacterial diseases [11,12]. It is well known that a successful infection of a host bacteria depends on the recognition of specific host cell components by phage proteins, so-called bacterial receptor molecules [13,14]. Currently, the receptors of phages in Gram-negative bacteria can be divided into three categories, namely LPS, protein, as well as flagella and fimbriae receptors. It is well established that LPS plays an important role in bacterial virulence, but is also involved in bacterial susceptibility to phages [15,16]. This suggests a potential correlation between the two biological processes in pathogenic bacteria, the capacity to infect but also to be infected [17].

The coevolution of plant bacteria, phages, and plants created an interdependent network. Many studies have been carried out to explore the adaptation of Gram-negative pathogenic bacteria to ecological niches, host plants, and phages [3,18,19]. Interestingly, studies also found that the reduced virulence was often observed in phage-resistant strains of animal and human pathogenic bacteria, indicating a correlation between bacterial virulence to host plants and bacterial sensitivity to its phages [20,21]. The resistance of bacterial pathogens to phages may be mainly attributed to the prevention of phage adsorption, blocking DNA entry, and the CRISPR/Cas and modification-restriction systems. However, the molecular mechanisms for the co-evolution of Xoo with its phages and host plants are not fully understood.

The aim of this study was to determine the genes involved in both bacterial pathogenicity to rice plants and bacterial sensitivity to its phage by constructing a Tn5 transposon mutant library, and by the generation and complementation of gene knockout mutants of Xoo, as well as analysis of bacterial phenotypes such as phage sensitivity and the pathogenicity to rice. The results of this study will provide the basis to understand the co-evolution of Xoo with both phages and host rice plants.

## 2. Materials and Methods

### 2.1. Bacterial Strains and Growth Conditions

Strain C2 used in this study was isolated from rice leaves infected with bacterial blight in Zhejiang Province by our laboratory and was identified as *Xanthomonas oryzae* pv. *oryzae* (Xoo) based on the analysis of 16S rDNA sequence, while the genome of strain C2 has also been sequenced (data not shown). Unless specified, Xoo strains were routinely grown in nutrient agar (NA) medium (1% tryptone, 0.5% glucose, 1% NaCl, 0.3% beef extract, and 1.5% agar, pH 6.8) at 30 ℃, while *Escherichia coli* strains were grown on Luria–Bertani (LB) medium (1% tryptone, 0.5% yeast extract, 1% NaCl, and 1.5% agar, pH 6.8) at 37 °C. When required, media were supplemented with antibiotics as follows: 10 μg/mL chloromycetin (Chl) and 20 μg/mL kanamycin (Km).

### 2.2. Bacteriophage and Plasmids

The phage X2 that was able to infect strain C2 was isolated as described in our previous study by using the Xoo pathogen as the target [18]. To construct knockout mutants, the suicide plasmid pJP5603 [22] was used, while the broad-spectrum host expression vector pRADK [23] was used to construct the corresponding complemented strains. All bacterial strains, plasmids, mutants, and complements used in this study (Table 1) were stored in the −80℃ refrigerator in our laboratory.

### 2.3. Phage Infection Assay

Phage infection was determined based on the method of the double plate, which was carried out as described by Ogunyemi et al. [18]. In brief, a single bacterial colony was picked and inoculated into 5 mL NB containing 20 μg/mL of Km. After overnight incubation at 30 °C with shaking at 200 rpm/min, 1 mL of bacterial culture with 0.6 of OD_600_ was added to 5.0 mL NA (0.7% agar) medium containing 20 μg/mL Km, and then overlaid on NA (1.5% agar) containing 20 μg/mL of Km. Following the inoculation with 5 μL of X2 phage, the double plates were incubated at 30 °C for 48 h to observe plaques.

### 2.4. Construction of Tn5 Mutants Library

Tn5 mutants library of Xoo strain C2 was constructed in this study by using the transposase–transposon complex (EZ::TN^TM^ < KAN-2 > Tnp Transposome^TM^ kit), which was carried out following the manufacturer’s manual (Epicentre, Madison, WI, USA). In brief, aliquots of 40 μL of the overnight incubated cells were mixed with 20 ng of EZ:TN^T^ AN-2 > Tnp Transposome. The cells were recovered by incubation at 30 °C for 3 h in 1 mL nutrient broth (NB) following electroporation in 0.2 cm cuvettes at 1.8 kV, 24 μS, and 200 Ω. After that, the transformants were inoculated on NA plates containing 20 μg/mL of Km and incubated at 30 °C for 3–4 d. Finally, single Km-resistant colonies were individually numbered and transformed into new NA plates with 20 μg/mL of Km for further investigation.

### 2.5. Identification of the Insertion Site of the Tn5 Mutants

The genomic DNA of the Tn5 mutants was extracted by using the bacterial genomic DNA extraction kit (Songon Biotech, Shanghai, China). In brief, the genomic DNA was firstly digested by restriction endonuclease *Bam*HI, ligated by T4 ligase, and then reverse PCR amplification with specific primers (Table 2). The insertion site of the Tn5 mutants was identified by sequencing the PCR product and then blasting with the strain C2 genome and NCBI database, while the names, the CDS sequence numbers, predicted functions, the sequence length, and E Value of each gene were recorded based on the analysis of bioinformatics analysis.

### 2.6. Construction and Complementation of Knockout Mutants

The knockout mutants of Xoo were generated as described by Liu et al. [24], based on the method of homologous recombination, which was carried out by electroporating the suicide plasmid pJP5603 carrying the target fragment into cells of strain C2 and then spreading on the NA medium containing 20 μg/mL of Km. Furthermore, the complemented strains were generated by electroporating the expression vector pRADK carrying the full-length gene fragment into the corresponding gene knockout mutant and then spreading on the NA medium containing 20 μg/mL of Km and 10 μg/mL of Chl. Primers for construction and complementation of the knockout mutants are listed in Table 3. In addition, to justify the identity of each strain, we analyzed the 16S rDNA sequence of the obtained knockout mutants and the complemented strains, which were performed as described by Zhang et al. [25].

### 2.7. Pathogenicity Assays in Rice Seedlings

Bacterial pathogenicity to rice seedlings was evaluated using the two methods, including spot infiltration and leaf clipping, which were carried out on susceptible rice variety “Xiangliangyou 900”. After centrifuging the overnight culture in NB containing 20 μg/mL of Km and then re-suspending with sterile water, the final concentration of bacterial suspension was adjusted to OD_600_ = 1.0. Spot infiltration was performed by inoculating 100 μL of bacterial suspension in leaves of 2-week-old rice seedlings with needleless syringes, while the pathogenicity to rice seedlings was determined by measuring the size of the lesions after 7 d of inoculation. Leaf clipping was performed by using scissors to dip bacterial suspension, then cutting off the 2-cm long part of the tip of the flag leaf from one-month-old rice seedlings. The pathogenicity to rice seedlings was determined by measuring the lesion length after 14 d of inoculation. The experiment was repeated in triplicate and each treatment had at least five replicates.

### 2.8. Motility and Biofilm Formation Assay

Motility was determined by measuring the diameter of the swimming zones according to the method of Ahmed et al. [26], which was performed by spotting 5 μL of overnight bacterial culture with a 0.5 of OD_600_ on the semi-solid NA (0.7% agar) medium containing 20 μg/mL of Km (if necessary) and then incubating the NA plates at 30 °C for 72 h. Biofilm formation was determined as described by Ahmed et al. [27], according to the method of the crystal violet by measuring the absorbance at the wavelength of 570 nm, which was carried out on 96-well microplate by inoculating each well with 200 μL of overnight bacterial culture with a 0.5 of OD_600_ and then incubating the microplate at 30 °C for 72 h. The experiment was performed three times and each treatment had three biological replicates.

### 2.9. Statistical Analysis

All experiments were done using a completely randomized design and the results were expressed as mean of three replicates (*n* = 3) ± standard deviation. Statistical analysis was performed using the SPSS software package 16.0 version (SPSS Inc., Chicago, IL, USA). The variations between the groups were estimated using the analysis of the different test. The results were statistically significant when the value was *p* < 0.05.

## 3. Results

### 3.1. Screening for Genes Responsible for Phage Resistance Using a Tn5 Mutant Library

**Figure 1:**
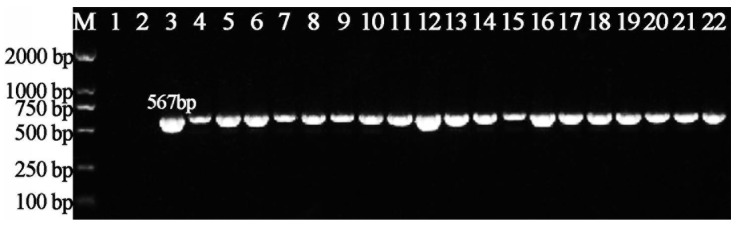
PCR amplification of bacteria from Tn5 mutant library according to the existence of Kan^R^ gene on EZ-Tn5 but not in the wild type of *Xanthomonas oryzae* pv. *oryzae* strain C2. M: Maker, DL2000; 1: H_2_O; 2: WT, Wild Type; 3: positive control pRADK; 4~22: Tn5 mutants.

**Figure 2:**
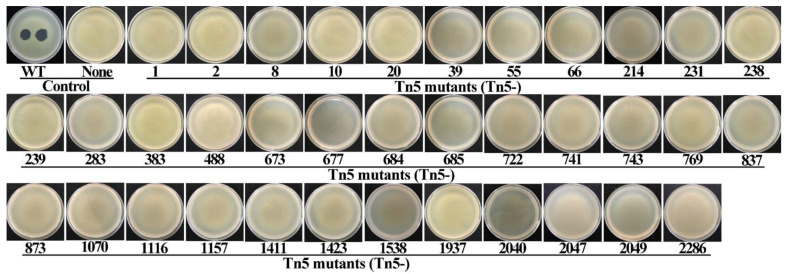
Screening of phage-resistant mutants by evaluating the infection of phage X2 to the constructed 4524 Tn5 mutants of *Xanthomonas oryzae* pv. *oryzae* strain C2. WT: Wild type (+phage); None: wild type (-phage); Tn5-(number): 36 phage-resistant Tn5 mutants (no plaques).

In order to identify genes that result in a bacterial phenotype that cannot be infected, we used the method of Tn5 transposon insertion. To this end, we constructed a comprehensive transposon mutant library, which consisted of a total of 4524 mutants of Xoo strain C2. Phage-resistant mutants were identified by the absence of a plaque formation (Figure 1). An infection by phage X2 results in the formation of plaques on a double-layer agar plate containing Xoo strain C2. Such plaques were not observed in 36 of the 4524 Tn5 mutants, demonstrating that these mutants were resistant to the infection of phage X2. The successful insertion of the Tn5 transposon into the genome of the phage-resistant mutants was verified by PCR amplification using a pair of specific primers (Table 2). All of the Tn5 mutants contained only single Tn5 insertions (data not shown). Figure 2 shows the PCR amplicons of 19 phage-resistant Tn5 mutants representing transposon insertion into different genes (described in the following section).

### 3.2. Identification of Genes That Confer Phage Resistance

**Figure 3:**
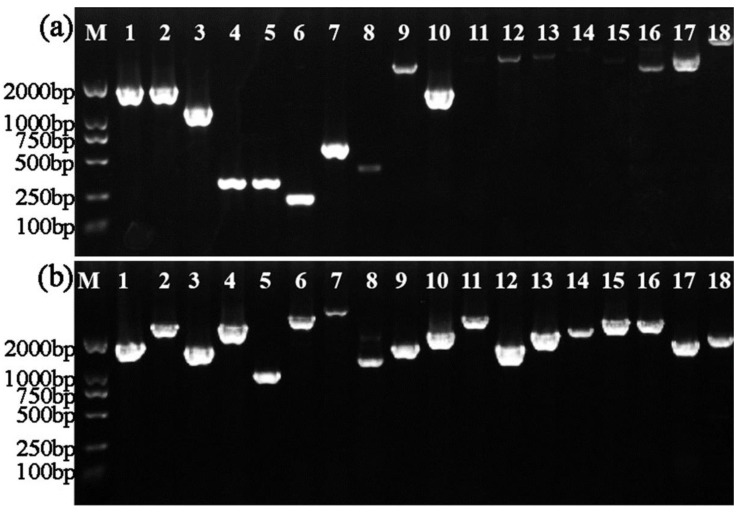
A single band with the expected size ranging from 176 bp to 4803 bp was observed in agarose gel electrophoresis results of inverse PCR amplification of the 36 phage-resistant Tn5 mutants. (**a**) M: Marker, DL2000; 1~18: Tn5-(66, 214, 231, 2, 383, 488, 1423, 8741, 1937, 1157, 39, 1538, 2040, 722, 1070, 2049, and 2286). (**b**) M: Marker, DL2000; 1~18: Tn5-(1, 10, 20, 55, 238, 239, 283, 673, 677, 684, 685, 743, 769, 837, 873, 1116, 1411, and 2047).

The insertion sites of the 36 phage-resistant Tn5 mutants were determined by inverse PCR amplification using specific primers (Table 3). Each mutant produced a single DNA band with sizes ranging from 176 bp to 4803 bp (Figure 3). The Tn5 insertion sites were determined by aligning the flanking sequences with strain C2 genome, which showed 98.31–100% homology (Table 1) with the corresponding CDS sequence as the C2 genome has not been deposited in a database. The sites of the Tn5 transposon insertion of the 36 mutants were located in 19 different genes as in some of the mutants the transposon inserted into the same gene. For the subsequent experiments, only one mutant for each gene was selected. The sequence length, name, and predicted function of each gene are listed in Table 1.

### 3.3. Functional Prediction of Phage-Resistance Mediating Genes

Bioinformatic analysis revealed that several genes are known receptors in other bacteria, such as genes involved in the LPS synthesis pathway, while others were novel or of unknown function. Table _X_ lists all genes that resulted in a phage resistant phenotype. One gene encodes nucleoside-diphosphate-sugar epimerases (Tn5-1157), UDP-2,3- diacylglucosamine diphosphatase (Tn5-1423), lipopolysaccharide core biosynthesis protein (Tn5-1070), glycosyl transferase (Tn5-488), the twin-arginine translocation pathway signal protein (Tn5-1538), transposase (Tn5-231), electron transfer flavoprotein beta subunit (Tn5-10), putative glycosyl/glycerophosphate transferases (Tn5-8), putative O-antigen acetylase (Tn5-283), cystathionine beta-synthase (Tn5-238), glycosyl transferase (Tn5-20/677/684/769/837), cystathionine gamma-synthase (Tn5-2/383), dTDP-glucose 4, 6-dehydratase (Tn5-39/55), glucose-1-phosphate thymidylyltransferase (Tn5-66/214/1/673/743/1411/2049), NAD dependent dehydrase (Tn5-741/2047), hypothetical protein (gene C2_803; Tn5-722/685/873/1116), hypothetical protein (gene C2_820, Tn5-2040/2286), hypothetical protein (gene C2_797, Tn5-239), and hypothetical protein (C2_802, Tn5-1937), respectively. Notably, the functions of the last four hypothesized proteins, which were encoded by different genes, are still unclear.

### 3.4. Verification of Phage Resistance by Knockout Mutation and Complementation

**Figure 4:**
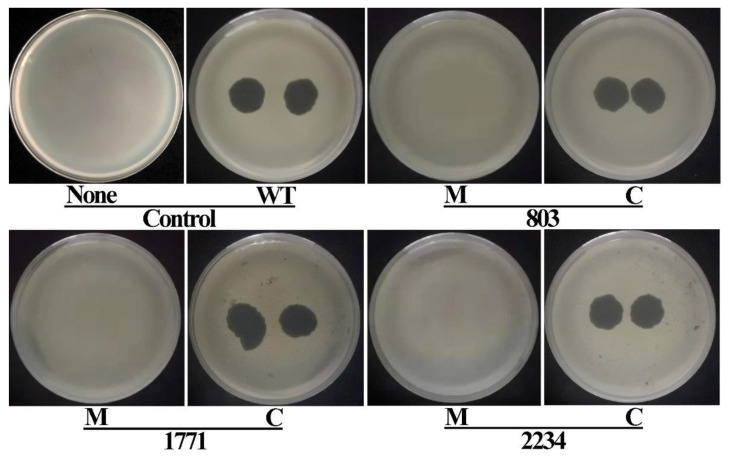
Verification of phage resistance by constructing the knockout mutants and complemented strains of the corresponding location genes of the 3 Tn5 mutants M488, M722 and M1070. WT: Wild type (+phage); None: wild type (−phage); M: Knockout mutant; C: Complemented strains.

The roles of genes mediating resistance to phage X2 were confirmed by constructing knockout mutants of the corresponding genes of the three Tn5 mutants M488, M722, and M1070, using homologous recombination. Identical to the result observed with the Tn5 insertion mutations, no plaque was observed in double-layer agar plates when performing the experiment with the three knockout mutants (Figure 4). When complementing the knocked-out genes in trans (via a plasmid), we were able to observe plaques, demonstrating that the disruption of removal of the genes results in phage resistance, while the presence allows the bacterium to be infected by X2.

### 3.5. Pathogenicity Analysis of Tn5 Mutants

**Figure 5:**
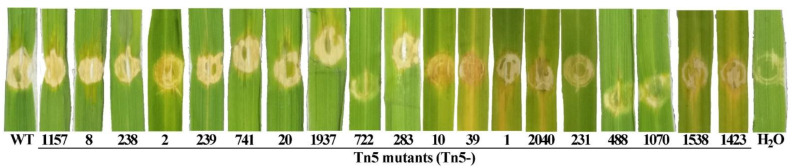
Effect of Tn5 insertion mutation on bacterial pathogenicity to rice were determined by infiltration inoculation of 19 Tn5 mutants representative of different genes on rice leaves. WT: Wild type strain C2; H_2_O: Negative control.

Although the genes were identified to convey resistance to the phage, we wondered which effect the disruption of the gene would have on the bacterial pathogenicity to infect rice. To this end, we randomly selected one Tn5 mutant from each of the 19 genes for a spot infiltration test of rice leaves. As shown in Figure 5, there was no major difference in the virulence of the 16 mutants compared to the wild type. Almost all strains, wild type and mutants, caused a dark-yellow spot on rice leaves after 7 days post-inoculation, with the exception of three Tn5 mutants M488, M722, and M1070. Here, the staining was less pronounced and the pattern more similar to the negative control (water). The three mutants that can be considered less virulent in the assay, the Tn5 insertion occurred in genes encoding glycosyltransferase (M488), a hypothetical protein (M722), and lipopolysaccharide core biosynthesis protein (M1070), respectively (Table 1).

### 3.6. Verification of Pathogenicity Attenuation by Knockout Mutation and Complementation

**Figure 6:**
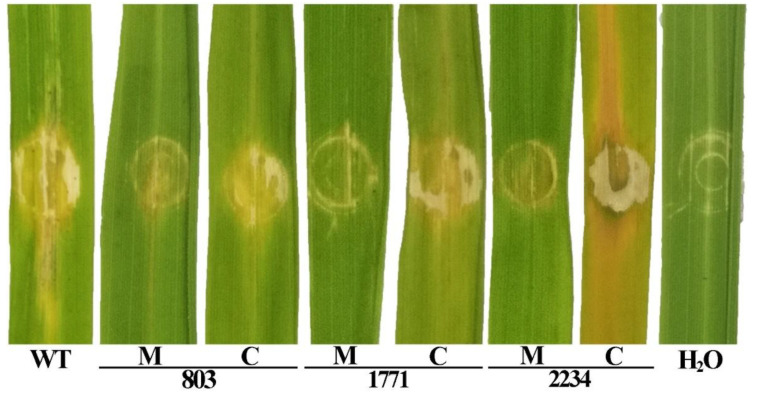
Influence of the 3 Tn5 mutants M488, M722 and M1070 in pathogenicity was justified by infiltration inoculation of knockout mutants and complemented strains of the corresponding location genes on rice leaves. WT: Wild type; H_2_O: Negative control; M: Knockout mutant; C: Complemented strains.

We also evaluated the pathogenicity of the knockout mutants and the complemented strains containing the plasmid-encoded genes in trans. Tests were performed on rice seedlings using the spot infiltration assay as well as the leaf clipping method. As shown in Figure 6, the spot infiltration inoculation of the wild type and the complemented strains caused dark-yellow spots around the inoculation area.

**Figure 7:**
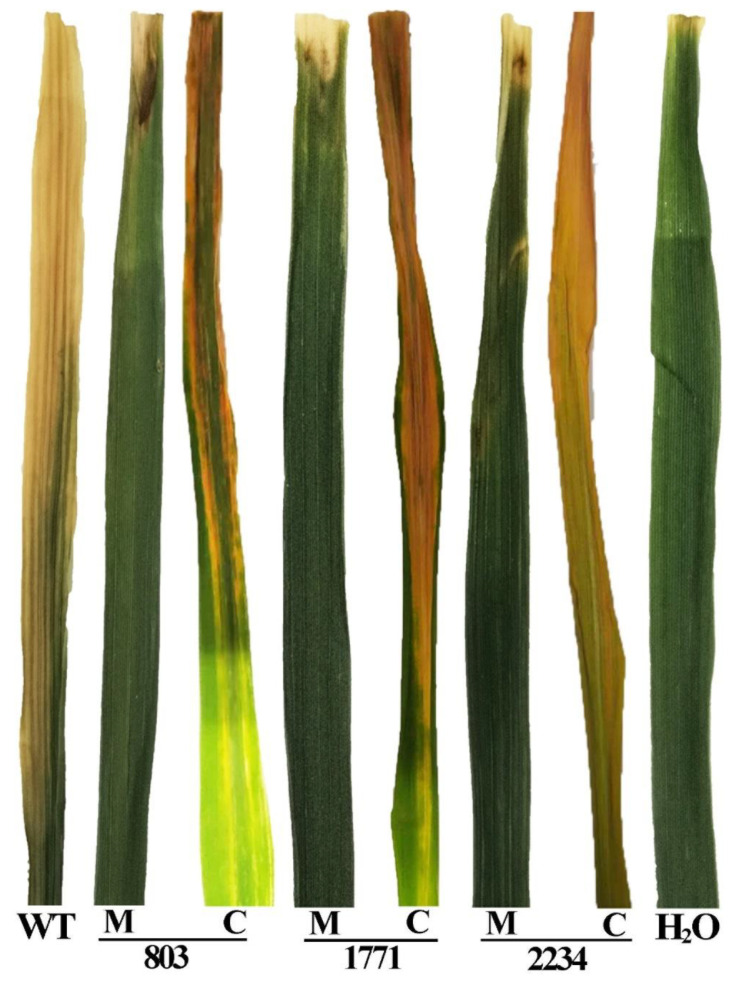
Influence of the 3 Tn5 mutants M488, M722, and M1070 in pathogenicity was demonstrated by leaf clipping inoculation of knockout mutants and complemented strains of the corresponding location genes on rice leaves. WT: Wild type; H_2_O: Negative control; M: Knockout mutant; C: Complemented strains.

**Figure 8:**
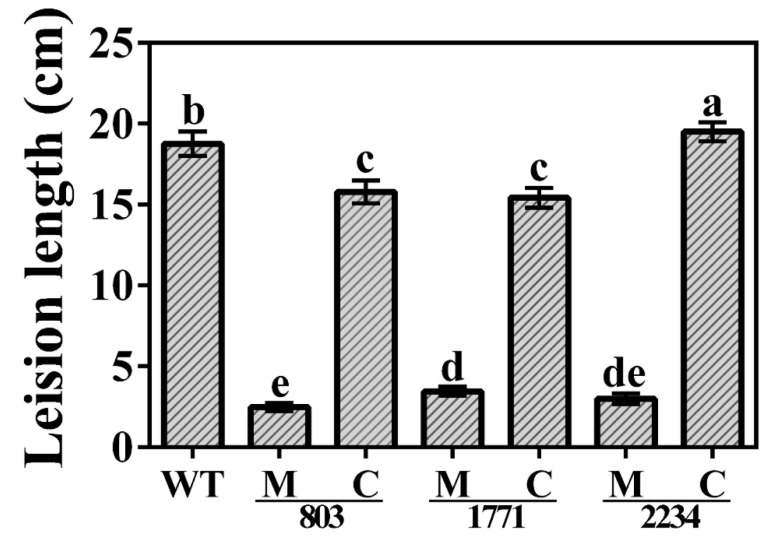
Lesions length on leaves of rice at 14 days after clipping inoculation of the three knockout mutants and corresponding complemented strains of *Xanthomonas oryzae* pv. *oryzae* strain C2. WT: Wild type; H_2_O: Negative control; M: Knockout mutant; C: Complemented strains. Lesion length (cm) was presented as means ± standard errors (*n* = 3). Columns with different letters (a–e) are significantly different according to LSD test (*p* = 0.05).

The inoculation with the knockout mutants Δ803, Δ1771 and Δ2234 resulted in a visible reduction in pathogenicity to the rice leaves as compared with the wild type strain. Here, the inoculation area was covered by only a slight spot with no expansion. The result of the leaf clipping assay showed a similar trend in bacterial pathogenicity. As shown in Figure 7 and Figure 8, yellowish-brown lesions were initially observed at the inoculation point and then spread across the whole rice leaves up to a length of ~19 cm following day 14 post inoculation with the wild type strain C2. Bacterial pathogenicity to the rice seedlings was significantly attenuated in the knockout mutants Δ803, Δ1771, and Δ2234, which caused a ~87%, ~82%, and ~84% reduction in lesion length compared to the wild type. When the complemented knockout strains were used, bacterial pathogenicity to rice seedlings was restored to ~84%, ~82%, and ~104%, respectively.

### 3.7. Effects of Gene Knockout on Motility and Biofilm Formation

**Figure 9:**
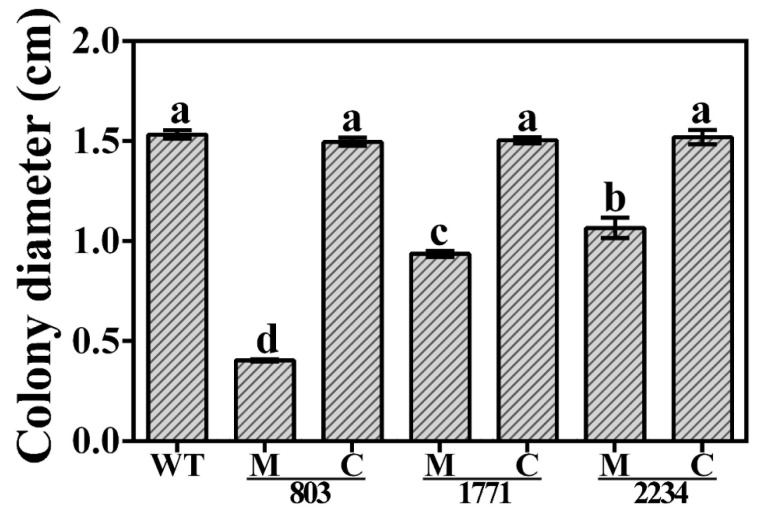
Motility of the three knockout mutants and corresponding complemented strains of *Xanthomonas oryzae* pv. *oryzae* strain C2. WT: Wild type; M: Knockout mutant; C: Complemented strains. Colony diameter (cm) was presented as means ± standard errors (*n* = 3). Columns with different letters (a–d) are significantly different according to LSD test (*p* = 0.05).

Aside from effects of the gene disruption or deletion on virulence of the bacterium, we also investigated the consequences on the motility of Xoo strain C2. We observed that the motility was significantly reduced in all three knockout mutants, with a ~74%, ~39% and ~30% reduction in the colony diameter of the mutants Δ803, Δ1771 and Δ2234, compared to the wild type. When complementing in trans, motility was effectively restored. Here, the complementation caused a ~271%, ~60% and ~42% increase in colony diameter compared to the corresponding knockout mutants and a similar level in motility compared to the wild type (Figure 9).

**Figure 10:**
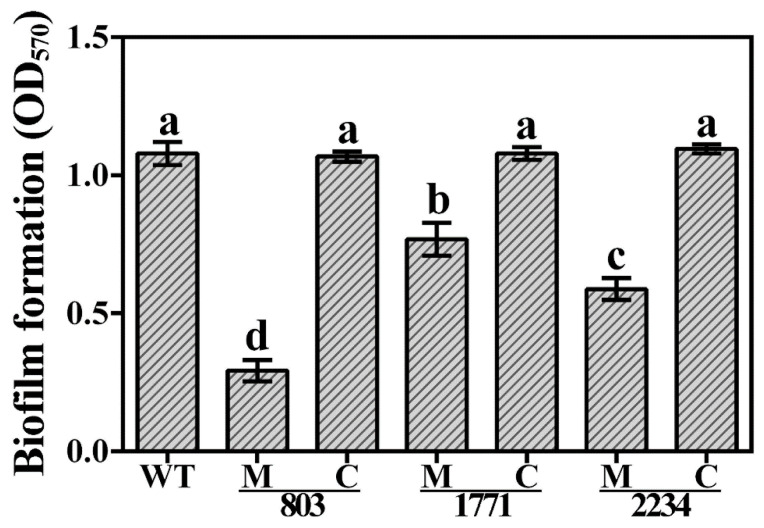
Biofilm formation of the three knockout mutants and corresponding complemented strains of *Xanthomonas oryzae* pv. *oryzae* strain C2. WT: Wild type; M: Knockout mutant; C: Complemented strains. Data of optical density at 570 nm (OD570) were presented as means ± standard errors (*n* = 3). Columns with different letters (a–d) are significantly different according to LSD test (*p* = 0.05).

In addition to motility, we also investigated the effect on biofilm formation. Compared to the wild-type strain, Xoo strain C2, the ability of the knockout mutants to form biofilms was significantly reduced but was effectively restored by complementation. We observed a ~73%, ~29%, and ~46% reduction in biofilm production in the mutants Δ803, Δ1771 and Δ2234, respectively. The complementation restored the capability of the strains to form biofilms to a similar extent as is the case for the wild type (Figure 10).

## 4. Discussion

In our study, we identified 19 bacterial mutants of the rice pathogen *Xanthomonas oryzae* pv. *oryzae* (Xoo) that conferred resistance to phage X2, using a Tn5 random transposon insertion resulting in a library of 4524 mutants. We calculated a frequency of Tn5 insertion of 52.78% in our study. Based on our whole genome sequencing data that revealed a Xoo C2 genome size of 4.94 Mbp, with 4943 ORFs (data not shown), we would require about 8571 mutants to screen the complete genome of this bacterium comprehensively. However, we only obtained 4524 mutants. However, many genes might be essential for the bacterium and disruption by Tn5 would be lethal. Nevertheless, further genes that mediate phage resistance might exist.

While 16 of the 19 phage-resistant mutants displayed similar pathogenicity towards rice plants compared with the wild type, three appeared to be less virulent. We confirmed the role of the three genes in phage resistance and bacterial pathogenicity by constructing knockout mutants as well as complementing them in trans from plasmid encoded genes. The three genes (*rfb303* and two unidentified genes) code for a protein involved in the biosynthesis of LPS. It is well established that LPS, long chains of sugars connected to a lipid core, can play a role in both virulence and phage attachment [28,29]. Interestingly, among the 19 genes associated with phage infection, 12 genes, accounting for nearly two-thirds of the identified genes, including *cysB*, *metB*, *wxoD*, *rmlB*, *rmlA*, *rfb303*, and *lpxH*, have been found to be involved in the LPS synthesis and transport [30,31,32]. For example, Perry et al. [16] reported that adsorption of ΦV10 and other phages can be blocked by *wxoD* encoding putative O-antigen acetylase by modifying the O157 antigen of bacterial LPS. Furthermore, Eugster et al. [33] reported the genes *rmlB* and *rmlA* are associated with bacterial resistance to phages by blocking adsorption of T7 phage to the lipid A portion of the LPS. In addition, [15] reported that lipid A of the LPS encoded by gene *lpxH* played an important role in binding of T-even phages.

We also observed a negative correlation of three phage-infection-associated genes with bacterial pathogenicity to rice seedlings. The three genes encode glycosyl transferase of the LPS core region, a hypothetical protein, and a LPS core biosynthesis protein, which has been reported to be involved in LPS synthesis and transport [32]. Similar to our observations, Petrocelli et al. [34] demonstrated the involvement of a glycosyl transferase encoded by gene *rfb303* (XAC2294) in bacterial virulence, which influenced motility and biofilm formation in *Xanthomonas axonopodis* pv. *citri*. Pérez-Pascual et al. [35] reported that the glycosyl transferase was important in the physiology and virulence of *Flavobacterium psychrophilum*.

A correlation between bacterial virulence and phage resistance has been reported recently by Alseth et al. [20], who reported that the virulence of *Pseudomonas aeruginosa* was weakened when the bacteria evolved a resistance mechanism to a phage infection which was mediated by a change of the bacterial envelope. In our study, the role of the three LPS-related genes in the pathogenicity of Xoo to rice seedlings could explain the fact that LPS is involved in bacterial virulence in various plant pathogenic bacteria, including Xoo [3]. In addition, the involvement of the three LPS-related genes in bacterial pathogenicity may be, at least partially, due to the indirect inhibition in twitching motility and biofilm formation.

The results of this study also provide a new insight for our understanding of the interaction of bacteria with phages and host (here: the rice plant). Indeed, phages have been used to control several plant diseases [36,37,38]. Yet, bacteria have evolved various mechanisms such as the inhibition of phage adsorption to protect themselves from the continuous attack of the natural enemy phage [39,40,41]. This, however, seems to result in a reduced level of virulence (or: fitness) at least in some cases. This may indicate that using phages as biocontrol agents might not only be effective due to the direct effect of killing bacteria, but also due to an indirect effect by attenuating bacterial virulence. Interestingly, recent studies have found that the emergence of phage-resistance in different bacteria can be prevented by using bacteriophage cocktails [42].

## 5. Conclusions

This study revealed a possible correlation between the bacterial ability to display resistance to a prey, the bacteriophage, while in turn losing fitness displayed in the reduction in virulence, loss of motility and capability to form biofilms. Using a Tn5 transposon mutant library, we identified phage-resistant mutants of Xoo C2, which we confirmed by constructing knockouts and plasmid complementations. Of the identified 19 genes, 12 are involved in LPS synthesis and transport, and three genes were also found to be involved in bacterial pathogenicity. Overall, our findings reveal the basis to understand the co-evolution of Xoo with both phages and host rice plants. However, this research area is still in infancy, and plenty of pioneering studies are required to fully explore the deployment of phages for plant disease management.

## Figures and Tables

**Table 1:** Identification of the Tn5 insertion site by using reverse PCR.

Mutants	CDS no. (%Sequence Similarity)	Functional Prediction of Genes		
		Name	Length	Description
Tn5-(1, 66, 214, 673, 743, 1411, 2049)	C2_813 (98.31–100)	*rmlA*	888	glucose-1-phosphate thymidylyltransferase
Tn5-(2/383)	C2_795 (98.48, 100)	*metB*	1194	cystathionine gamma-synthase
Tn5-8	C2_601 (98.63)	*tagB*	1056	putative glycosyl/glycerophosphate transferases
Tn5-10	C2_811 (100)	*etfB*	747	electron transfer flavoprotein beta subunit
Tn5-(20, 677, 684, 769, 837)	C2_801 (98.94–100)	―	711	glycosyl transferase
Tn5-(39, 55)	C2_812 (98.83/100)	*rmlB*	975	dTDP-glucose 4, 6-dehydratase
Tn5-231	C2_918 (99.54)	―	99	Transposase
Tn5-238	C2_794 (99.88)	*cysB*	1377	cystathionine beta-synthase
Tn5-239	C2_797 (99.56)	―	801	Hypothetical protein
Tn5-283	C2_804 (99.65)	*wxoD*	336	putative O-antigen acetylase
Tn5-488	C2_1771 (100)	―	2100	glycosyl transferase
Tn5-(722, 685, 873, 1116)	C2_803 (99.85–100)	―	243	Hypothetical protein
Tn5-(741, 2047)	C2_798 (98.66, 99.95)	―	462	NAD dependent dehydrase
Tn5-1070	C2_2234 (98.83)	*rfb303*	969	lipopolysaccharide core biosynthesis protein
Tn5-1157	C2_68 (100)	―	681	Predicted nucleoside-diphosphate-sugar epimerases
Tn5-1423	C2_3900 (98.66)	*lpxH*	743	UDP-2,3- diacylglucosamine diphosphatase
Tn5-1538	C2_3261 (100)	―	2328	The twin-arginine translocation (Tat) pathway signal protein
Tn5-1937	C2_802 (99.65)	―	291	Hypothetical protein
Tn5-(2040, 2286)	C2_820 (99.71/99.89)	―	2088	Hypothetical protein

**Table 2:** List of plasmids and bacterial strains used in this study.

Strains	Relevant Characteristics	References
*X. oryzae pv oryzae* strains		
C2	wild type, the pathogen of rice bacterial blight	In our lab
*C2_803*	KmR; C2 insertional mutant defective in C2_803	This study
*C2_1771*	KmR; C2 insertional mutant defective in C2_1771	This study
*C2_2234*	KmR; C2 insertional mutant defective in C2_2234	This study
*C2_803-comp*	ChlR, KmR; complement strain of C2_803	This study
*C2_1771-comp*	ChlR, KmR; complement strain of C2_1771	This study
*C2_2234-comp*	ChlR, KmR; complement strain of C2_2234	This study
phage X2 Xoo	Isolated from pathogen of rice bacterial blight	In our lab
*E. coli* strains		
DH5α	Recombinant plasmid replication	Qingke Hangzhou
S17-1 λpir	Recombinant plasmid replication	Simon et al. (1983)
pJP5603 plasmid and its derivatives		
pJP5603	Suicide plasmid, Km^R^	Penfold et al. (1992)
pJP5603-803	Km^R^, pJP5603 with *C2_803* flanking fragment	This study
pJP5603-1771	Km^R^, pJP5603 with *C2_1771* flanking fragment	This study
pJP5603-2234	Km^R^, pJP5603 with *C2_2234* flanking fragment	This study
Plasmids vector pRADK and its derivatives		
pRADK	Amp^R^, Chl^R^, Kan^R^, the broad-spectrum host expression vector	Gao et al. (2005)
pRADK-803	Amp^R^, Chl^R^, Kan^R^, pRADK with *C2_*803 flanking fragment	This study
pRADK-1771	Amp^R^, Chl^R^, Kan^R^, pRADK with *C2_*1771 flanking fragment	This study
pRADK-2234	Amp^R^, Chl^R^, Kan^R^, pRADK with *C2_*2234 flanking fragment	This study

**Table 3:** List of primers used in this paper.

Primers	Primer Sequences (5′-3′)	Targeted Genes	
		Name	Length (bp)
Kan-F	AAGGTAGCGTTGCCAATGAT	Kan^R^	567
Kan-R	GCCGTTTCTGTAATGAAGGA		
KAN-2 FP-1	ACCTACAACAAAGCTCTCATCAACC	Kan^R^	162
KAN-2 RP-1	GCAATGTAACATCAGAGATTTTGAG		
803-F	CGGGATCCGCGTGGCTATTGCGAGGAG	C2_803	217
803-R	CGGAATTCGTGCACAAACGCTTCTACCTG		
1771-F	CGGGATCCGCGTCCTGATACAGACGCAC	C2_1771	1538
1771-R	CGGAATTCGTGCTGCTCAACAACGACAC		
2234-F	CGGGATCCGTGCGTGCTGCACCTGCTG	*rfb303*	968
2234-R	CGGAATTCCAGGAATTGCGCAGTCCATCG		
803-C-F	GAGCTCGAATTCTAGACGGTGTATAACGGGCGGG	C2_803	604
803-C-R	GCAGAAGCTTCTAGAGTGCACAAACGCTTCTACCTG		
1771-C-F	GAGCTCGAATTCTAGACTTGCACAACGCTGGGTTG	C2_1771	2602
1771-C-R	GCAGAAGCTTCTAGACCAACGCAATGTCGATGTC		
2234-C-F	GAGCTCGAATTCTAGAGGAAGGGCGCATCGAAGC	*rfb303*	1475
2234-C-R	GCAGAAGCTTCTAGACAGGAATTGCGCAGTCCATCG		

## Data Availability

Not applicable.
